# Assessing probe-specific dye and slide biases in two-color microarray data

**DOI:** 10.1186/1471-2105-9-314

**Published:** 2008-07-19

**Authors:** Ruixiao Lu, Geun-Cheol Lee, Michael Shultz, Chris Dardick, Kihong Jung, Jirapa Phetsom, Yi Jia, Robert H Rice, Zelanna Goldberg, Patrick S Schnable, Pamela Ronald, David M Rocke

**Affiliations:** 1 Department of Data Analysis and Algorithm, Affymetrix, Inc., Santa Clara, California, USA; 2 College of Business Administration, Konkuk University, Korea; 3 Department of Molecular Biosciences, University of California, Davis, California, USA; 4 Department of Plant Pathology, University of California, Davis, California, USA; 5 Center for Plant Genomics, Iowa State University, Ames, Iowa, USA; 6 Department of Environmental Toxicology, University of California, Davis, California, USA; 7 Department of Radiation Oncology, University of California, Davis, Cancer Center, Sacramento, California, USA; 8 Division of Biostatistics, University of California, Davis, California, USA

## Abstract

**Background:**

A primary reason for using two-color microarrays is that the use of two samples labeled with different dyes on the same slide, that bind to probes on the same spot, is supposed to adjust for many factors that introduce noise and errors into the analysis. Most users assume that any differences between the dyes can be adjusted out by standard methods of normalization, so that measures such as log ratios on the same slide are reliable measures of comparative expression. However, even after the normalization, there are still probe specific dye and slide variation among the data. We define a method to quantify the amount of the dye-by-probe and slide-by-probe interaction. This serves as a diagnostic, both visual and numeric, of the existence of probe-specific dye bias. We show how this improved the performance of two-color array analysis for arrays for genomic analysis of biological samples ranging from rice to human tissue.

**Results:**

We develop a procedure for quantifying the extent of probe-specific dye and slide bias in two-color microarrays. The primary output is a graphical diagnostic of the extent of the bias which called ECDF (Empirical Cumulative Distribution Function), though numerical results are also obtained.

**Conclusion:**

We show that the dye and slide biases were high for human and rice genomic arrays in two gene expression facilities, even after the standard intensity-based normalization, and describe how this diagnostic allowed the problems causing the probe-specific bias to be addressed, and resulted in important improvements in performance. The R package LMGene which contains the method described in this paper has been available to download from Bioconductor.

## Background

One of the major tasks in the analysis of high-dimensional biological assay data such as gene expression arrays is to detect differential expression from a comparative experiment. Using two-color microarrays is supposed to adjust for the noise introduced by many factors on the same slide including spot size and conformation. Standard data pre-processing methods for two-color data include the normalization of the differences between two dye channels, after which most users believe the dye bias has effectively been removed and that the normalized measurements are now relatively free of dye bias. However, probe specific dye-bias and slide-bias can be high even after standard normalization, which may cause problems when one expects to identify many statistically significantly differentially expressed genes.

This dye bias has received some recent attention [1-8]. These papers generally provide computational methods to detect and correct for dye bias, at least in some circumstances. Correction can include use of gene-specific dye bias terms in an ANOVA, for example. Even when this is done, dye bias may still cause significant harm by introducing large amounts of noise that prevent identification of significantly differentially expressed genes. We present a graphical method of assessing this problem that can be used for process improvement and to compare array platforms.

Standard normalization methods are based on the entire set of probe intensities of the arrays, while the conclusions of comparative experiments are made for specific probes. One of the common approaches for the analysis is gene-by-gene linear models, which uses the normalized log or glog [9] intensity data and is fitted for each probe. In the routine gene-by-gene linear model, the mean square (MS) of each factor is the measurement of the variance contribution from the factor, which is also the base of the construction of F-statistic for testing the factor effect. So, for each probe, the relative sizes of the mean squares can serve as comparison measures of the contributions of the specific factors to the overall variation.

For the standard F statistic, we consider the ratios of each mean square to an appropriate error term, which is usually also a mean square. We propose instead as a diagnostic to consider the ratio of each mean square to the sum of all the mean squares, so that we obtain for each gene a set of mean-square ratios that sum to 1, which are thus free of scaling specific to a given probe. To assess the overall magnitudes of these quantities, we plot the empirical cumulative distribution functions (ECDF) of the variability proportion of each factor across the whole set of probes in a single plot, serving as the diagnostic graphic tool for showing the relative magnitude of the probe specific dye-bias after normalization. Since the linear model is on a probe-by-probe basis, the dye bias we are measuring is in fact the dye-by-probe interaction. Similarly, including slide as one of the factors could also provide an assessment of the relative size of the slide-by-probe interaction effect. The lower a line is in the plot, the larger the effect's mean square is stochastically across probes.

## Results and methods

In most cases being shown in this paper, the linear model, including factors of interest dye, slide, treatment and sample replicates, can be written as:

(1) *y*_*ijkl *_= *α *+ dye_*i *_+ slide_*j *_+ treat_*k *_+ sample_*l *_+ *ϵ*_*ijkl*_,

where the index *i *refers to different channels (dyes), the index *j *to arrays, the index *k *to treatment levels and the index *l *to the sample replicates within each treatment level [10].

Consider as a first example an experiment conducted on slides spotted and hybridized at a UC Davis array facility. The experimental objective was to study the effects of oxygen concentration on gene expression before and at confluence in human keratinocyte cell cultures. There were three different oxygen concentrations used, with two replicates in each condition. Labeled sample was hybridized with common reference for each condition. In each case, in one of the replicates the sample was labeled with Cy3 and the reference with Cy5, and in the other replicate the reverse labeling was used.

**Figure 1:**
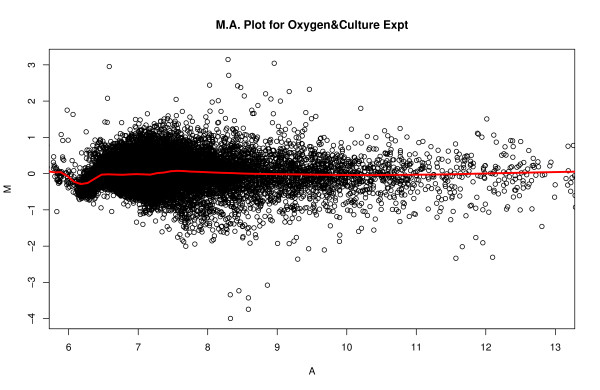
MA Plot of oxygen concentration experiment, run by UCD array facility.

**Figure 2:**
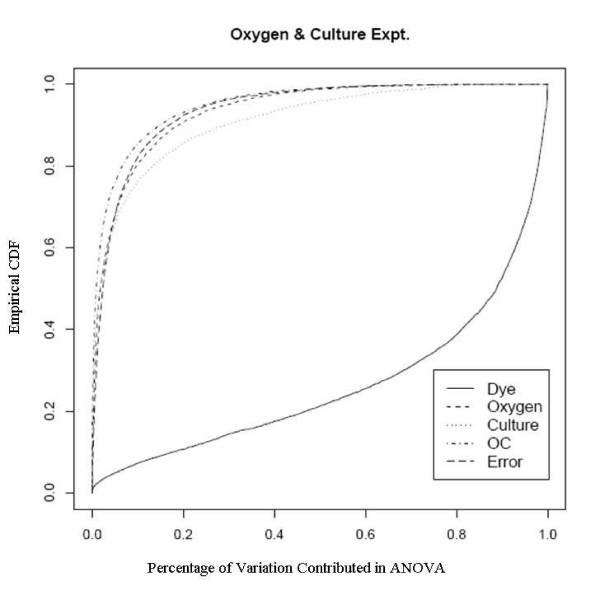
Empirical CDF Plot of oxygen concentration experiment, run by UCD array facility.

The MA plot [11,12], where M is the difference between the probe or probe set log intensity in Cy3 and Cy5 channels, and A is the average of the probe or probe set log intensity in the two channels, could demonstrate if the data set has intensity-dependent log ratios. From the MA plot in Figure 1, we can see that, after the normalization, most of the dye bias has been removed. However, when we look at the average mean squares of ANOVA model on a probe-by-probe base, the probe-specific dye factor by far contributes the most variation in the model, as shown in the Table 1, either before normalization or after normalization. The ECDF plot, which defined in Background section and in which lower line demonstrates larger effect, shows that the probe specific dye effect, after normalization, is also the largest factor, same as shown by the average mean squares from ANOVA, and is much larger than the treatment effects of oxygen and culture conditions (Figure 2). The substantial probe-dye bias is an obstacle to detection of significantly expressed genes, and not surprisingly, few of the probes show significant differential expression for different oxygen and culture levels. In this case, we have used a model in which the log ratio of sample to reference is given by a linear model involving dye, oxygen, culture, and the oxygen-by-culture interaction. Note that, due to the reference design used, the slide effect cannot be estimated here. This would, given the characteristics of the experiment, be slide nested within oxygen and culture, and this is confounded with dye nested in the same way.

**Table 1:** Average Mean Squares from ANOVA for Oxygen Experiment

	Dye	Oxygen	Culture	Oxy:Cul Interaction	Residual
Before Normalization	16.58	0.0358	0.0836	0.0256	0.0938
After Normalization	1.730	0.0246	0.0565	0.0196	0.0209

A second example shows a comparison of the analysis of the same RNA on two different two-color array platforms. The samples were from human skin biopsies exposed in vivo to controlled radiation doses incidental to radiation therapy, but with accurate dosimetry [13,14]. Patients were treated in a standard fashion for their localized prostate cancer and the areas of their abdominal wall skin which would receive 1, 10, 100 cGy of radiation exposure respectively were marked at the time of the patient's first radiation treatment. Prior to any radiation therapy, patients had a control biopsy, at 0 dose. In this component of the study, there were 8 patients, and the data for the array comparison are the four samples from patient 5. The samples were run on two different array platforms: arrays spotted by a UC Davis array facility and Agilent Human Whole Genome arrays run by Icoria's Paradigm Array Labs. The model used had log intensity as a linear function of dose, or else of modified log dose, which was -1, 0, 1, and 2 for the doses 0, 1, 10, and 100. This is log_10 _dose except that the 0 dose is treated as if it were 0.1 cGy. We call this modified log dose or mld.

For the arrays from the UC Davis facility, we used a design in which each dose was hybridized against each other dose. With dye swaps, this would have required 12 arrays, but we instead used a partial balance of the dyes against the treatments. The exact design is given in Table 2. For the Agilent arrays, we used the design in Table 3.

**Table 2:** Experimental Design for IR Study with UC Davis Arrays

Channel	Array1	Array2	Array3	Array4	Array5	Array6
Red	A	A	D	B	B	C
Green	B	C	A	C	D	D

A = 0 cGy, B = 1 cGy, C = 10 cGy, D = 100 cGy

**Table 3:** Experimental Design for IR Study with Agilent Arrays

Channel	Array1	Array2	Array3	Array4
Red	A	D	C	B
Green	D	A	B	C

A = 0 cGy, B = 1 cGy, C = 10 cGy, D = 100 cGy

Some care must be taken in the analysis of these data. Unlike a reference design study, we are not analyzing the log ratios. Instead, we analyze the separate values for each gene on each array and each dye channel. There is only one biological sample for each dose, and the variation between replicate measurements of the same RNA is not an appropriate denominator for a test of significance of the regression. We could specify this as a mixed model, with separate random effects for the sample (with 4 levels) and replicates within sample, but fitting such models by maximum likelihood results in many estimation failures using standard software because the model must be fitted for each gene of thousands. Instead, we first fit a model with dose or mld as a quantitative variable, then fit another model with dose or mld as a factor. Then we use the decrease in the residuals sum of squares of the first model to that of the second model. We quantify the within-sample variation in this alternative way and this gives us two degrees of freedom.

**Figure 3:**
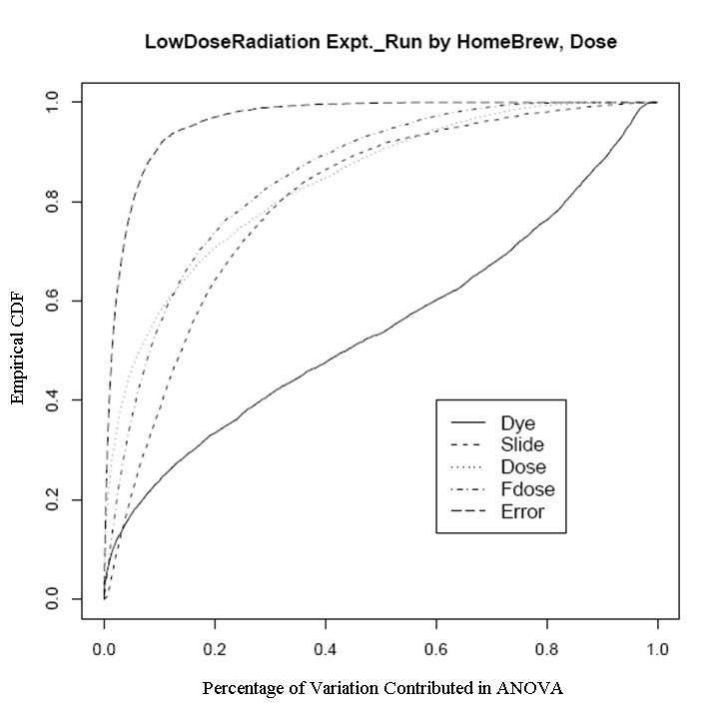
Empirical CDF Plot of Low Dose Radiation study with linear dose, run by UCD array facility.

**Figure 4:**
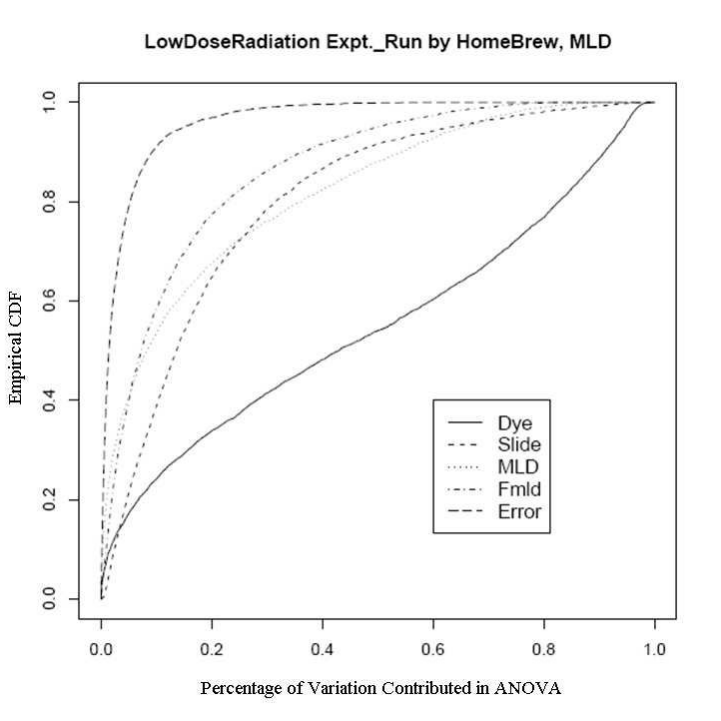
Empirical CDF Plot of Low Dose Radiation study with modified log-linear dose, run by UCD array facility.

**Figure 5:**
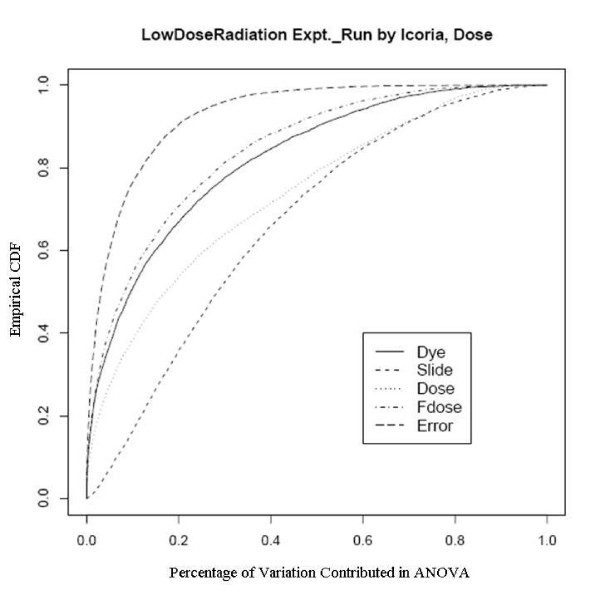
Empirical CDF Plot of Low Dose Radiation study with linear dose, run by Icoria.

**Figure 6:**
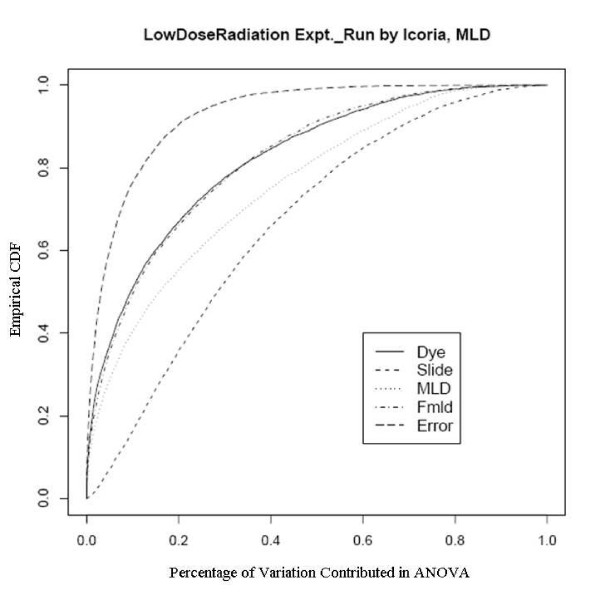
Empirical CDF Plot of Low Dose Radiation study with modified log-linear dose, run by Icoria.

After an empirical Bayes adjustment of the denominators and based on the FDR p-values (Rocke 2004), we obtained the numbers of significant genes from the two platforms as shown in Table 4. We can see that the number of significant genes is much larger for the Agilent platform even though the number of arrays is smaller. Since the RNA is the same and the technology is similar, there must be a quality issue explaining the difference. We show the diagnostic plots for dye and slide bias for the two platforms in Figures 3 and 4 for the UC Davis spotted arrays, and Figures 5 and 6 for the Agilent arrays, with the two plots being for the regression on dose and mld respectively. The ECDF plots for the custom arrays tell that even after lowess normalization, the differences between two dye channels per probe base still contributes the most variability among all the factors, for both linear dose and log-linear dose cases. Slide-by-probe interactions are also rather large. For the Agilent arrays, the slide-by-probe interaction has the largest variance. The dose effect is the second, yet comparable to the first, and larger than the probe specific dye bias. This is the likely reason why we obtained more significantly differentially expressed probes from the Agilent arrays. These examples suggested that there were problems in the UC Davis array facility, and this guided improvements in the process that greatly reduced the probe-dye bias problem, as subsequent examples show.

**Table 4:** Significant Genes from Two Platforms

Facility	30% FDR	20% FDR	10% FDR	5% FDR
UC Davis	0	0	0	0
UC Davis	38	4	0	0
Agilent	4445	3119	1912	1367
Agilent	1553	1018	0	0

In either case in UC Davis or Agilent, the numbers on the first row are the number of significant genes for the regression on dose and the numbers on the second row are for the regression on modified log dose.

**Figure 7:**
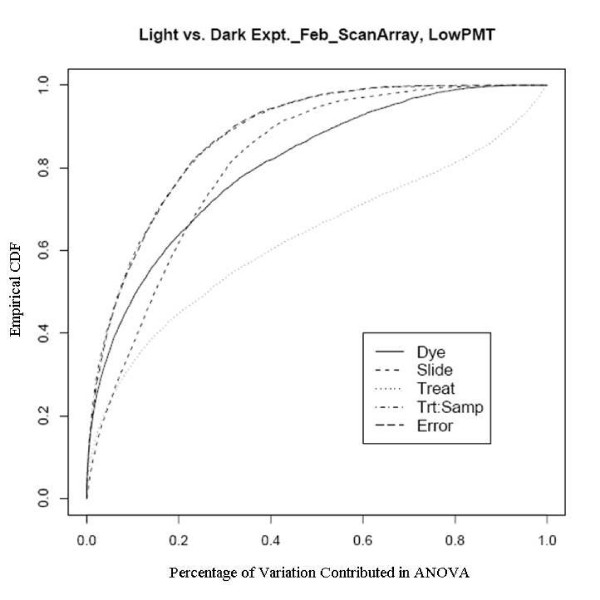
Empirical CDF Plot of rice genome light and dark experiment run in Feb., using ScanArray scanner with low PMT, run by UCD array facility.

**Figure 8:**
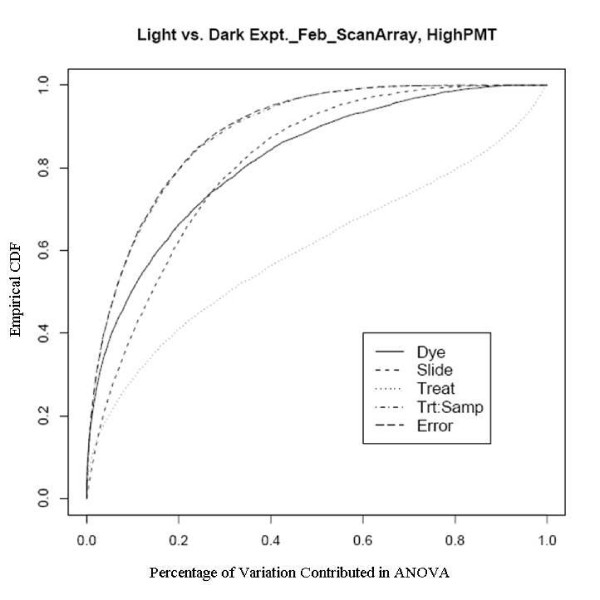
Empirical CDF Plot of rice genome light and dark experiment run in Feb., using ScanArray scanner with high PMT, run by UCD array facility.

**Figure 9:**
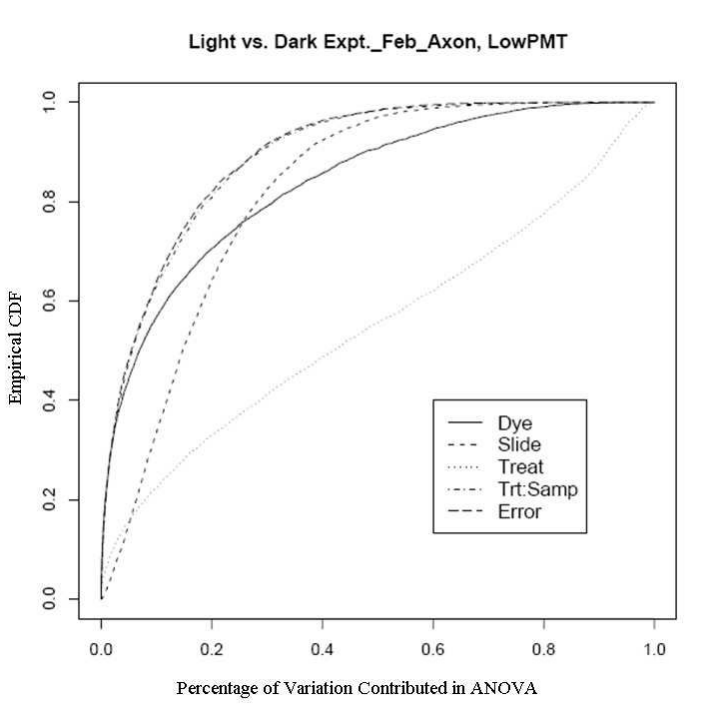
Empirical CDF Plot of rice genome light and dark experiment run in Feb., using Axon scanner with low PMT, run by UCD array facility.

**Figure 10:**
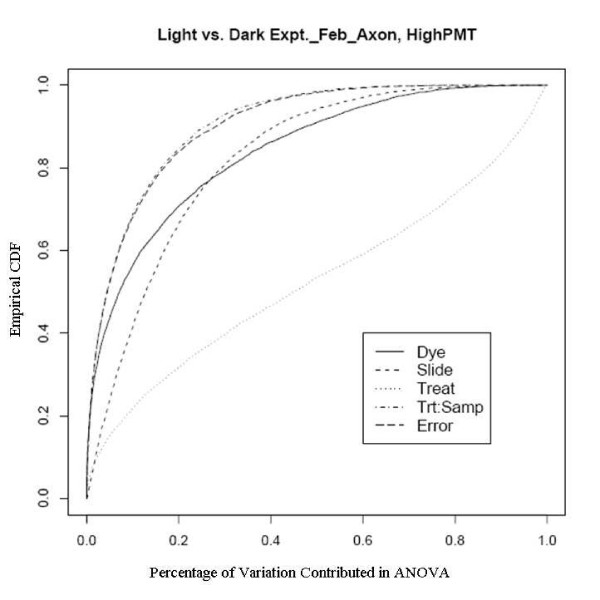
Empirical CDF Plot of rice genome light and dark experiment run in Feb., using Axon scanner with high PMT, run by UCD array facility.

**Figure 11:**
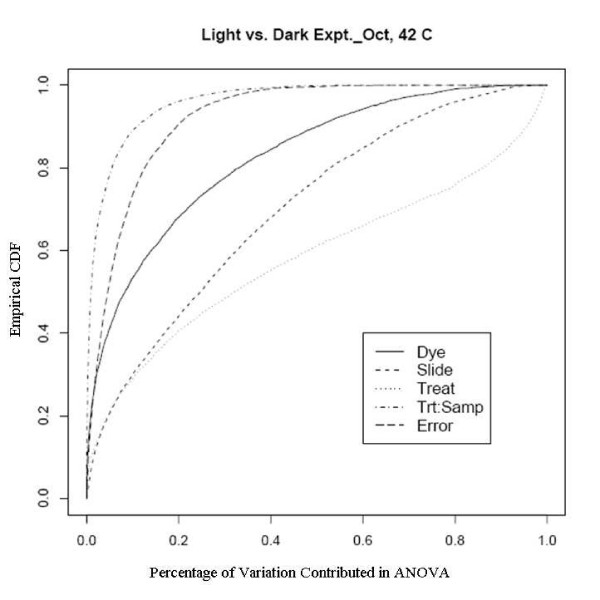
Empirical CDF Plot of rice genome light and dark experiment run in Oct., at temperature of 42, run by UCD array facility.

**Figure 12:**
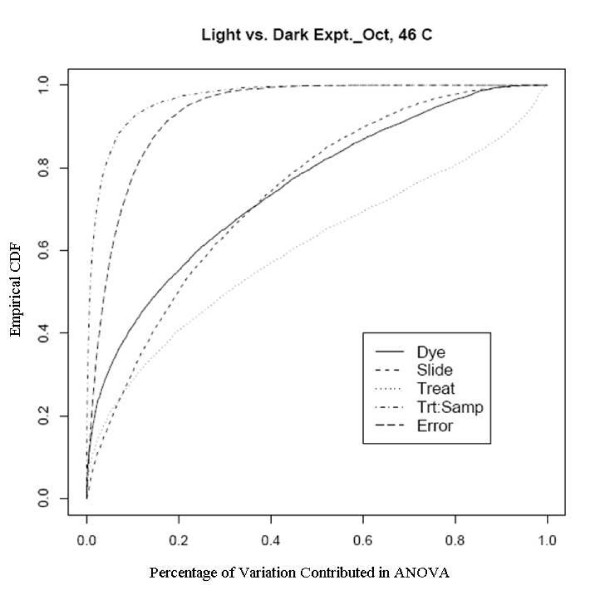
Empirical CDF Plot of rice genome light and dark experiment run in Oct., at temperature of 46, run by UCD array facility.

**Figure 13:**
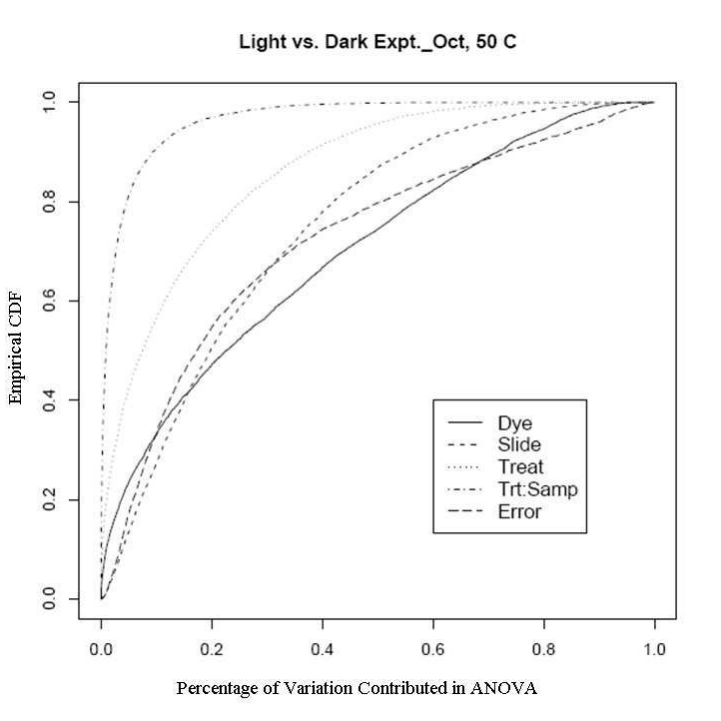
Empirical CDF Plot of rice genome light and dark experiment run in Oct., at temperature of 50, run by UCD array facility.

An example showing this improvement comes from an experiment using a newly developed rice genome array in which rice plants grown in the dark were compared with those grown under normal lighting conditions. This relatively extreme treatment was used specifically to evaluate the dye and slide bias, given that the expression changes to the treatments should be large. There are two sets of experiments used here, one was done in February 2005 to evaluate the effect of different scanners and different scanner settings on dye and slide bias and the other was in October 2005 to assess the effects of temperature on the biases. The experiments in February were run on two different scanners with two different PMT (Photo Multiplier Tube) levels. Figure 7, 8, 9 and 10 clearly show that after the normalization, the factors dye and slide do not exceed the influence from the factor treatment per probe, which means that these biases are not likely to interfere with detection of significant differential expression. The experiments in October were done at three different culture temperature levels. From the ECDF plots (Figure 11 and 12) for the first two lower temperature (42C and 46C), we can see that the factor treatment is the most influential one, while the factor dye and slide become the biggest ones in the case of 50C (Figure 13), suggesting that the lower temperatures are likely to be superior.

## Conclusion

Most microarray users assume that any differences between the dyes that may cause problems in an analysis can be handled by standard methods of normalization. However, there are still probe specific dye bias and slide bias afterwards in two-color microarray data. We developed a procedure for quantifying the extent of them. The primary graphical diagnostic was used to show the probe-specific dye and slide bias exist and can be quite large after normalization in arrays from rice and humans, in two facilities. This tool guided improvements in the array facility at UC Davis that essentially eliminated the problematic dye bias behavior.

## Authors' contributions

RL, G–CL and DMR developed the statistical methodology. MS, YJ, RHR, ZG, PSS, PR, CD, KJ and JP conducted the microarray gene expression experiments and provided the data. All authors read and approve the manuscript.
